# Using Social Media for the Promotion of Education and Consultation in Adolescents Who Have Undergone Kidney Transplant: Protocol for a Randomized Control Trial

**DOI:** 10.2196/resprot.8065

**Published:** 2018-01-09

**Authors:** Claudiana Pase, Andréia Dias Mathias, Clotilde Druck Garcia, Clarissa Garcia Rodrigues

**Affiliations:** ^1^ Fundação Universitária de Cardiologia Programa de Pós Graduação Instituto de Cardiologia Porto Alegre Brazil; ^2^ Hospital da Criança Santo Antônio Irmandade Santa Casa de Misericórdia Porto Alegre Brazil; ^3^ Serviço de Nefrologia Pediátrica Hospital da Criança Santo Antônio Irmandade Santa Casa de Misericórdia Porto Alegre Brazil

**Keywords:** transplantation, education, social network, adolescents

## Abstract

**Background:**

Falling ill represents a traumatic experience especially in adolescence, since in addition to the moments of ambiguity and contradictions that this period brings, there is coping with the disease. Renal transplantation provides a better quality of life but the dependence on dialysis is replaced by the greater responsibility of self-care. With advances in technology, contemporary communication methods are a strategic mechanism for the approximation of the adolescent and the multiprofessional team. In this perspective, our research may provide possible changes and propose alternatives, using social networks for the integration of the multiprofessional team, promoting education within a virtual environment for adolescents who have undergone kidney transplants.

**Objective:**

The goal of our research is to compare the knowledge, satisfaction, and self-esteem of adolescent renal transplant patients in 2 groups: patients undergoing conventional treatment versus patients undergoing conventional treatment plus the full-time use of social networks to aid in education and consultation.

**Methods:**

Nonblind randomized clinical trial with 128 adolescents (aged 13 to 21 years) divided in 2 groups: the first group will receive conventional care and the second group will be invited to participate in a secret group on the social network Facebook. This group will be used as a new education platform to involve young renal transplant patients to participate in the guidelines provided to them by the multiprofessional team.

**Results:**

An environment for learning and exchanging life experiences will be created by using a well-known technology among adolescents. As a low-cost intervention, it will allow a better interaction between the patient and the transplant team. It is expected that the adolescents will improve their knowledge about the disease also increasing their self-esteem and the treatment adhesion.

**Conclusions:**

Health professionals need to seek alternatives when educating patients, focusing on easily understandable ways for effective guidance. In the adolescent population, it is understood that the use of technology as support in education is a fundamental tool for this age group. The proposed project will directly benefit adolescent renal transplant patients as it uses language aimed directly at the target demographic. It attempts to overcome the traditional model by being more in contact with the current generation. This approach makes the content easier to assimilate and, consequently, increases understanding.

**Trial Registration:**

ClinicalTrials.gov: NCT03214965; https://clinicaltrials.gov/ct2/show/NCT02239354 (Archived by Webcite at http://www.webcitation.org/6wKnYrFGx)

## Introduction

### Background

Chronic renal failure is a disease of major impact on the lives of adolescents, with a high morbidity and mortality. It is a worldwide public health problem, and renal transplantation is a therapeutic alternative [1]. In Brazil, the pediatric population is approximately 53 million (according to data by the Brazilian Institute of Geography and Statistics). Brazil has a prevalence of 20 terminal chronic kidney disease cases per million of population and an incidence of 6.6 per million of population [1,2].

According to the Brazilian Association of Organ Transplant, it is estimated that 349 pediatric patients per year are registered on the kidney list. In 2015, 396 children joined the list, and 316 had transplants. The number of kidney transplants for children has changed every year. In 2014, there was an increase of 13.3% compared to 2013. The next year, however, the 532 organ transplants represented a drop of 5% in relation to 2014 [1,2].

Adolescence is a period of life when there are several physiological and morphological changes. It is also the transition phase to adulthood, represented by the struggle for independence and the pursuit for personal identity. Falling ill represents a traumatic experience especially in adolescence, since in addition to the moments of ambiguity and contradictions that this period brings, there is coping with the disease. An important adolescent characteristic is impulsive thought added to the difficulty of predicting the consequences of their actions, relevant factors for the health professional to include in the guidelines for the public of this age [3,4].

The above mentioned factors can compromise adherence to treatment. This problem has been observed more frequently in adolescents aged 11 to 19 years with low self-esteem, depression, and lacking the support of at least 1 adult. Nonadherence to treatment is one of the major problems in adolescence, and the survival of the graft undergoes a sharp drop of 62% in 5 years and 36% in 10 years after transplantation [5,6,7].

Although transplanted adolescent patients are cared for by a multidisciplinary team that helps them face the situation of the disease, minimizing their anguish and orienting them to adapt to changes in their daily lives, we find it difficult to increase the participation of these patients.

With advances in technology, contemporary communication methods are a strategic mechanism for the approach of the adolescent and the multiprofessional team, reducing barriers for education using a language more characteristic of the age. The introduction of new technologies in education has been improving knowledge, in the sense that takes the form of a helpful didactic object in teaching, capable of generating and providing its users with scientific knowledge in a spontaneous manner [8]. Facebook is one of the most popular social networks in the world. It allows social interaction by exchanging and sharing posts, organizing users into groups, and providing an environment to discuss ideas. When used with a well-grounded knowledge source, Facebook may be able to improve the quality of health care [9].

In this perspective, our research may provide possible changes and propose alternatives by using social networks for the integration of the multiprofessional team, promoting education within a virtual environment for adolescents who have undergone kidney transplants. In this way, a means of reference capable of ensuring the expansion of care will be provided.

### Objectives

The goal of our research is to compare the knowledge, satisfaction, and self-esteem of adolescent renal transplant patients, gauged through questionnaires, of 2 groups: patients undergoing conventional treatment versus patients undergoing conventional treatment plus the full-time use of social networks to aid in education and consultation.

## Methods

### Study Design

This study is a nonblind randomized clinical trial. Facebook will be used as a new education platform to involve young renal transplant patients in participating in the guidelines provided to them by the multiprofessional team. It is believed that the use of this tool as an aid in the education of adolescents will cause an improvement in communication and interaction between patients and staff and promote a space for the exchange of experiences between patients of the same age. This will facilitate the expression of feelings and contribute to increased self-esteem, reduced anxiety, and increased adherence to the treatment plan. Considering that Facebook is an already used social network with worldwide coverage, use is possible regardless of location.

The study will be developed with patients from *Hospital da Criança Santo Antônio in the Sistema Única de Saude* (SUS) outpatient clinic. *Hospital da Criança Santo Antônio* is one of the 7 care units of Santa Casa de Porto Alegre.

The inclusion criteria are being between the ages of 13 and 21 years and being in treatment by the nephrology team in pre- and posttransplantation.

The exclusion criteria are patients in pretransplantation who are not registered on the state health department unique kidney transplant list and those who do not wish to participate in the study.

### Intervention

The adolescents will be invited to participate in the study during a routine visit to the outpatient clinic. Educational activities will be provided through the social network for a 3-month period by a multidisciplinary team comprising a nurse, doctor, psychologist, physical educator, social worker, and nutritionist.

The person responsible for the study will create an activity schedule for the team (Multimedia Appendix 1). Each week a professional will have the responsibility of creating educational material related to their specialty. The researcher will follow up with the adolescents daily for questions, answers, and encouragement to post on the social network. Playful and interactive materials will be provided facilitating the adolescent’s understanding.

At the beginning of the week, one of the professionals will launch a debatable theme for the week; this debate will be moderated by the author. The postings are of a purely informational nature and will conform to Conselho Federal de Medicina resolution 2133/2015 which, among other things, prohibits the carrying out of consultations, diagnostics, or prescriptions by any means of mass or distant communication [10].

In this study, a group will be created on Facebook. Facebook is a social network launched in 2004 that allows the connection between individuals by sharing their stories. Users customize their profiles that contain photos and lists of personal interests, exchanging messages, private and public, between themselves and participants from groups of friends [11].

To register on Facebook you must be older than 13 years and read and accept the statement of rights and responsibilities. It is prohibited to publish content or perform any act which infringes or contravenes the rights of third parties or the law; Facebook removes any content or information published if judged in violation of this statement or policies. When there are changes to these terms, users receive notifications and have the opportunity to review the revised terms.

On Facebook it is possible to create closed groups (ie, spaces dedicated to share updates, photos, or documents or send messages to a select group of people). Groups can be open, closed, or secret. In this study, we chose the secret group to bring more privacy; it functions as follows:

- Publications are visible only to members of the group
- An administrator is responsible for the creation and maintenance of the group who reviews the members’ posts and releases them for all to see
- Only a person invited to participate in the group may take part
- Only current members of the group can see who is in the group
- Only those who are included as part of the group may post comments, view the comments posted, or find the group on Facebook

### Control Group

Traditionally, all transplanted patients are treated as outpatients by a team of nephrologists. Individual consultations are carried out which provide guidance on treatment. Special attention is given to the adherence to the immunosuppressive drugs. The interval between appointments is usually 6 months or less according to clinical criteria. If required, advice is requested from other specialties.

### Randomization

Patients who are receiving medical follow-up treatment in the SUS outpatient clinic will be invited to participate in the study. Those who meet the eligibility criteria and wish to participate will receive a consent form to be completed by the person responsible for the adolescent and one to be completed by the adolescent. The next step will be the completion of the knowledge questionnaire, the satisfaction questionnaire, and the Rosenberg scale. Participants will be advised that the questionnaires will also be completed 3 months after the beginning of the study and that they will be sent a link by email.

The adolescents will be randomly selected by the program randomization.com, which will generate a list through randomization. The list will be held by a person outside the study responsible for sorting the participants according to the order they are drawn into intervention or control group. Study participants will be instructed on the dynamics of the draw, and if they are chosen to take part in the intervention group, the author will send an invitation through Facebook to join the group *Transplantados do Santo Antônio*.

### Outcome Measures

#### Knowledge

Participants will complete a 10-question multiple choice questionnaire (Multimedia Appendix 2) before and 3 months after the beginning of the study. The items in the questionnaire were taken from the agenda of a transplant patient (the agenda was created by a team of professionals who treat adolescent transplants), which is delivered by the medical team during the orientation consultations with the patient and family before transplantation. The agenda is used in the course of treatment, where the notes on the patient’s state of health after transplantation are kept.

#### Self-Esteem

The Rosenberg Scale (Multimedia Appendix 3) is used in order to evaluate self-esteem. It is based on the adaptation by Dini et al [12] for Brazil. It contains 10 items, 5 referring to a positive vision and 5 to a self-deprecating view of self, with answer choices strongly agree, agree, disagree, and strongly disagree. After completing the inversions and summing the answers, a score between 0 and 30 is obtained on the scale; the lower the score, the greater the level of self-esteem of the individual. The Rosenberg Scale is considered an adequate scale for the evaluation of self-esteem in adolescents because it is composed of a small number of items, uses simple language, and is fast and easy to score [13].

#### Satisfaction

A structured questionnaire with closed questions used to measure level of satisfaction (Multimedia Appendix 4) will be completed by the participant before and after the end of the study. This questionnaire contains 10 items rated on a 4-point Likert scale from totally unsatisfied to totally satisfied; the higher the score, the higher the level of satisfaction.

### Ethical Conditions

The study was approved by the Ethics Committee of the Institute of Cardiology/University Foundation of Cardiology and Hospital da Criança Santo Antônio. Before the adolescents can participate they will be given 2 copies of a consent form (for the adolescents) and 2 copies of an informed consent form (for the person responsible) in which consent for the participation in the study will be confirmed. The terms of the consent forms will be read together with the participants.

### Sample Size

The self-esteem results were used for the calculation of the sample due to a lack of studies for the other results. For the calculation of the sample, a significance level of 5% with a power of 80% was considered with a standard deviation of 6 points on the Rosenberg Scale that evaluates self-esteem, as in a study by Hutz and Zanon [14], with an expected difference of 3 points between the groups after the intervention, totaling a sample group of 122 participants (61 in each group).

### Statistical Analysis

A chi-square test will be used to analyze the statistics for the comparison of the groups.

## Results

An environment for learning and exchanging life experiences will be created by using a well-known social media technology among adolescents [15]. As a low-cost intervention, it will allow a better interaction between the patient and the transplant team. It is expected that the adolescents will improve their knowledge about the disease also increasing their self-esteem and the treatment adhesion.

## Discussion

### Summary

Innovation in health care doesn’t necessarily mean the development of a new technology or the use of a new method. To occur, innovation doesn’t need a huge amount of financial resources either. The health care professional can innovate simply by adopting small changes in a traditional process.

In our study, we are seeking a low-cost way to improve quality of life, treatment adhesion, and other aspects related to the follow-up of kidney transplanted adolescents. We decided to use Facebook (a free and well-known resource used by most of our patients daily) to transmit and share transplant-related knowledge, thus using a current technology as a new helper tool for these characteristic patients.

### Conclusion

Health professionals need to seek alternatives when educating patients, focusing on easily understandable ways for effective guidance. In the adolescent population, it is understood that the use of technology as support in education is a fundamental tool for this age group. The proposed project will directly benefit adolescent renal transplant patients as it uses language aimed directly at the target demographic. It attempts to overcome the traditional model by being more in contact with the current generation. This approach makes the content easier to assimilate and, consequently, increases understanding.

## Appendix

Multimedia Appendix 1 Activity schedule.

Multimedia Appendix 2 Evaluation questionnaire.

Multimedia Appendix 3 Rosenberg Self-Esteem Scale.

Multimedia Appendix 4 Satisfaction survey.

## Abbreviations

SUS: Sistema Única de Saude (public health system)

## References

[1] Garcia V, Pacheco L (2015). Dimensionamento dos transplantes no Brasil e em cada estado. Rev Bras Transpl.

[2] Sesso R (2002). Epidemiologia da Doença Renal Crônica no Brasil: Guia de Nefrologia.

[3] Morales L, Castilho E (2007). Vivencias de los(as) adolescentes en dialisis: una vida con multiples perdidas pero con esperanza. Colombia Med.

[4] Oliva M, Singh TP, Gauvreau K, Vanderpluym CJ, Bastardi HJ, Almond CS (2013). Impact of medication non-adherence on survival after pediatric heart transplantation in the USA. J Heart Lung Transplant.

[5] Najarian JS, Almond PS, Mauer M, Chavers B, Nevins T, Kashtan C, Matas AJ (1992). Renal transplantation in the first year of life: the treatment of choice for infants with end-stage renal disease. J Am Soc Nephrol.

[6] Pereira L (2012). Adesão ao Tratamento Imunossupressor no Transplante Renal.

[7] Silva DS, Livramento ML, Pereira LM, David NE (2009). Adesão ao tratamento imunossupressor no transplante renal. Braz J Nephrol.

[8] Garcez J, Maciel F, Cardoso V (2012). Considerações ergonômicas para aplicação de mídia em ambientes educacionais para crianças de ensino fundamental. Prod.

[9] Fumian A (2013). Novas Mídias: Facebook Como Ferramenta de Ensino em Ciência da Saúde [dissertação de mestrado].

[10] (2015). Resolução number 2133/2015.

[11] Ellison N, Steinfield C, Lampe C (2007). The benefits of Facebook friends: social capital and college students' use of online social network sites. J Comput-Mediated Communic.

[12] Dini G, Quaresma M, Ferreira L (2004). Adaptação cultural e validação da versão Brasileira da Escala de Auto-estima de Rosenberg. Rev Soc Bras Cir Plast.

[13] Lourenço L (2010). Short-form 36 e Escala de Autoestima Rosemberg-epm/unifesp em Paraplégicos com Ulcera por Pressão [dissertação de mestrado].

[14] Hutz C, Zanon C (2011). Revisão da adaptação, validação e normatização da Escala de Autoestima de Rosenberg. Aval Psicol.

[15] Leanza F, Hauser D (2014). Teens, technology, and health care. Prim Care.

