# Supplementary material for: Using Social Media for the Promotion of Education and Consultation in Adolescents Who Have Undergone Kidney Transplant: Protocol for a Randomized Control Trial
Source: JMIR Res Protoc. 2018 Jan 9;7(1):e3. doi: 10.2196/resprot.8065 (PMC5780617; doi:10.2196/resprot.8065)
Supplement: Multimedia Appendix 1 [file resprot_v7i1e3_app1.pdf]

Multimedia Appendix 1: ACTIVITY SCHEDULE

Week	Subject	Responsible
1	At the end of the day.... What do I have?	Nurse
2	Getting to know more about the transplant.	Nurse
3	Who is taking care of me?	Nurse
4	Eating for a transplant patient.	Nutritionist
5	Why me? Moment to work on feelings.	Psychologist
6	What I can do every day.	Physical Educator
7	And my future?	Doctor
8	Family moment	Psychologist
9	Rejection	Social Worker
10	Use of immunosuppressive drugs	Physician
11	Choosing the best path- Tips to maintain a healthy kidney	Nurse
12	I want to….I can	Psychologist
