# Supplementary material for: Using Social Media for the Promotion of Education and Consultation in Adolescents Who Have Undergone Kidney Transplant: Protocol for a Randomized Control Trial
Source: JMIR Res Protoc. 2018 Jan 9;7(1):e3. doi: 10.2196/resprot.8065 (PMC5780617; doi:10.2196/resprot.8065)
Supplement: Multimedia Appendix 2 [file resprot_v7i1e3_app2.pdf]

Multimedia Appendix 2: EVALUATION QUESTIONNAIRE

In accordance with post renal transplant care, answer the questions below:

1-What is a kidney transplant?
a) It is a surgery used to put a healthy kidney into my body.
b) It is the use of drugs to make the kidneys function correctly again
c) It is a probe inserted for the withdrawal of urine.
d) It is a set of blood tests.

2- After discharge from hospital, which is the care that I should have after a kidney transplant?
a) Take medication on time, not drinking water, blood pressure and weight control and physical exercise.
b) Avoid places with agglomerations of people, people with colds or flu, have a balanced diet, do not forget the medication times and follow all the guidelines given by the renal transplant team.
c) After a kidney transplant I am allowed to ingest any type of food, I cannot forget to take medication and I must have rigorous personal hygiene.
d) Strict control of blood pressure, do not have contact with other people and animals, physical exercise, do not ingest liquid and have dialysis.

3- Can I drink water after the transplant?
a) Drinking water is not allowed to so as not to overload the kidney.
b) After the transplant, it is important to drink plenty of water every day.
c) It should be ingested in moderation.
d) Only up to 3 glasses of water per day are allowed.

4- Should I avoid contact with animals?
a) In the first three months intimate contact with animals should be avoided.
b) I can have contact with animals, as long as they are clean.
c) Contact is allowed after the transplant, but with restrictions: only once a week.
d) It is extremely prohibited to have any contact with animals for the entire life after transplantation.

5- Can I perform physical activities?
a) I can perform all activities including: Boxing, Karate and Judo.
b) I shouldn’t do any activities so as not to hurt the kidney.
c) Only once per week to not have overload of the kidney.
d) Lower impact activities can be performed such as: swimming, hiking, biking and running.

6- What is rejection?
a) Rejection is when the kidney presents a problem because the person does not drink water.
b) When the kidney damages the body by working too hard.
c) It is when the body finds that the transplanted organ does not belong to it and tries to fight it;
d) Rejection is the term used to describe the reaction presented when a kidney has an infection.

7- How do I identify rejection?
a) Pain or swelling in the transplanted kidney; fever; decrease in urine; quick and large weight gain; swelling of eyelids, hands and feet; pain during urination; Increase in blood pressure;
b) Sore throat, increased urination, headache and increased appetite.
c) Abdominal pain, increased urination, swelling, weight gain and high blood pressure.
d) Urinary tract infection, increased appetite, weakness and increased urination.

8- Can I smoke or drink alcohol?
a) Allowed in moderation.
b) Not allowed because it can jeopardize my transplant.
c) Only at parties.
d) Only drinks with low alcohol content.

9- After transplantation for how long will I use medication (immunosuppressant)?
a) I must take medication for the rest my life.
b) Until the kidney is working well.
c) For the first two years.
d) Only when in pain.

10- What is the purpose of immunosuppressant?
a) To leave the kidney free of infections.
b) So that I don’t feel pain.
c) To hinder the entry of viral organisms.
d) It is the medication that prevents the rejection of my transplanted kidney.
