# Supplementary material for: Using Social Media for the Promotion of Education and Consultation in Adolescents Who Have Undergone Kidney Transplant: Protocol for a Randomized Control Trial
Source: JMIR Res Protoc. 2018 Jan 9;7(1):e3. doi: 10.2196/resprot.8065 (PMC5780617; doi:10.2196/resprot.8065)
Supplement: Multimedia Appendix 3 [file resprot_v7i1e3_app3.pdf]

Multimedia Appendix 3: THE ROSENBERG SELF-ESTEEM SCALE (RSS)

Below is a list of statements dealing with your general feelings about yourself. Please indicate how strongly you agree or disagree with each statement.

	Strongly Agree	Agree	Disagree	Strongly Disagree
1. On the whole, I am satisfied with myself.	☐	☐	☐	☐
2. At times I think I am no good at all. (unqualified or inferior to others)	☐	☐	☐	☐
3. I feel that I have a number of good qualities.	☐	☐	☐	☐
4. I am able to do things as well as most other people (as long as I am taught).	☐	☐	☐	☐
5. I am not satisfied with the things I have done. I feel I do not have much to be proud of.	☐	☐	☐	☐
6. I certainly feel useless at times (incapable of doing doings).	☐			
