# Supplementary material for: Using Social Media for the Promotion of Education and Consultation in Adolescents Who Have Undergone Kidney Transplant: Protocol for a Randomized Control Trial
Source: JMIR Res Protoc. 2018 Jan 9;7(1):e3. doi: 10.2196/resprot.8065 (PMC5780617; doi:10.2196/resprot.8065)
Supplement: Multimedia Appendix 4 [file resprot_v7i1e3_app4.pdf]

Multimedia Appendix 4: SATISFACTION SURVEY

Mark the alternative that is most relevant to your satisfaction with your treatment.

	**Totally Unsatisfied**	**Unsatisfied**	**Satisfied**	**Totally Satisfied**
1- Support by professionals during treatment.	☐	☐	☐	☐
2- Enough time to discuss what you would like to know with professionals.	☐	☐	☐	☐
3- Your knowledge about your disease and your treatment.	☐	☐	☐	☐
4- Your acceptance of the disease and treatment.	☐	☐	☐	☐
5- Autonomy allowed by the treatment.	☐	☐	☐	☐
6- Space to exchange experiences with other patients.	☐			
